# Supplementary material for: OpenMM 7: Rapid development of high performance algorithms for molecular dynamics
Source: PLoS Comput Biol. 2017 Jul 26;13(7):e1005659. doi: 10.1371/journal.pcbi.1005659 (PMC5549999; doi:10.1371/journal.pcbi.1005659)
Supplement: S1 Source Code — (ZIP) [file pcbi.1005659.s002.zip › OpenMM-7.0.1-Source/docs/api-c++/generated/OpenMM.AndersenThermostat.html]

AndersenThermostat — OpenMM @OPENMM\_MAJOR\_VERSION@.@OPENMM\_MINOR\_VERSION@ documentation


# AndersenThermostat¶

*class* `OpenMM::``AndersenThermostat`¶
:   This class uses the Andersen method to maintain constant temperature.

    Methods

    |  |  |
    | --- | --- |
    | `AndersenThermostat` | Create an `AndersenThermostat`. |
    | `getDefaultTemperature` | Get the default temperature of the heat bath (in Kelvin). |
    | `setDefaultTemperature` | Set the default temperature of the heat bath. |
    | `getDefaultCollisionFrequency` | Get the default collision frequency (in 1/ps). |
    | `setDefaultCollisionFrequency` | Set the default collision frequency. |
    | `getRandomNumberSeed` | Get the random number seed. |
    | `setRandomNumberSeed` | Set the random number seed. |
    | `usesPeriodicBoundaryConditions` | Returns whether or not this force makes use of periodic boundary conditions. |

    *const* std::string &`Temperature`()¶
    :   This is the name of the parameter which stores the current temperature of the heat bath (in Kelvin).

    *const* std::string &`CollisionFrequency`()¶
    :   This is the name of the parameter which store the current collision frequency (in 1/ps).

    `AndersenThermostat`(double *defaultTemperature*, double *defaultCollisionFrequency*)¶
    :   Create an `AndersenThermostat`.

        |  |  |
        | --- | --- |
        | Parameters: |  |

        - **defaultTemperature** – the default temperature of the heat bath (in Kelvin)
        - **defaultCollisionFrequency** – the default collision frequency (in 1/ps)

    double `getDefaultTemperature`() *const*¶
    :   Get the default temperature of the heat bath (in Kelvin).

        |  |  |
        | --- | --- |
        | Returns: | the default temperature of the heat bath, measured in Kelvin. |

    void `setDefaultTemperature`(double *temperature*)¶
    :   Set the default temperature of the heat bath. This will affect any new Contexts you create, but not ones that already exist.

        |  |  |
        | --- | --- |
        | Parameters: |  |

        - **temperature** – the default temperature of the heat bath (in Kelvin)

    double `getDefaultCollisionFrequency`() *const*¶
    :   Get the default collision frequency (in 1/ps).

        |  |  |
        | --- | --- |
        | Returns: | the default collision frequency, measured in 1/ps. |

    void `setDefaultCollisionFrequency`(double *frequency*)¶
    :   Set the default collision frequency. This will affect any new Contexts you create, but not ones that already exist.

        |  |  |
        | --- | --- |
        | Parameters: |  |

        - **frequency** – the default collision frequency (in 1/ps)

    int `getRandomNumberSeed`() *const*¶
    :   Get the random number seed. See `setRandomNumberSeed()` for details.

    void `setRandomNumberSeed`(int *seed*)¶
    :   Set the random number seed. The precise meaning of this parameter is undefined, and is left up to each `Platform` to interpret in an appropriate way. It is guaranteed that if two simulations are run with different random number seeds, the sequence of collisions will be different. On the other hand, no guarantees are made about the behavior of simulations that use the same seed. In particular, Platforms are permitted to use non-deterministic algorithms which produce different results on successive runs, even if those runs were initialized identically.

        If seed is set to 0 (which is the default value assigned), a unique seed is chosen when a `Context` is created from this `Force`. This is done to ensure that each `Context` receives unique random seeds without you needing to set them explicitly.

    bool `usesPeriodicBoundaryConditions`() *const*¶
    :   Returns whether or not this force makes use of periodic boundary conditions.

        |  |  |
        | --- | --- |
        | Returns: | true if force uses PBC and false otherwise |

High performance molecular simulation on GPUs


### Navigation

- Core Objects
- Forces
- Integrators
- Extras

©2015, Stanford University and the Authors.
|
Powered by Sphinx 1.3.1
& Alabaster 0.7.7
|
Page source
